# Comparative Assessment of Transcranial Doppler and MRI Perfusion Imaging in a Japanese Child With Probable Brain Death

**DOI:** 10.7759/cureus.99877

**Published:** 2025-12-22

**Authors:** Sho Kimura, Katsuhiro Abe, Kenta Ochiai, Taku Omata, Jun-ichi Takanashi

**Affiliations:** 1 Department of Pediatrics, Tokyo Women's Medical University Yachiyo Medical Center, Yachiyo, JPN; 2 Department of Pediatrics, Tokyo Women’s Medical University Yachiyo Medical Center, Yachiyo, JPN

**Keywords:** brain death, japanese, magnetic resonance imaging, pediatric, transcranial doppler

## Abstract

The determination of brain death differs internationally, although in Japan, it is legally recognized only when organ donation is intended. Otherwise, the condition is termed probable brain death. Ancillary tests, including transcranial Doppler (TCD) ultrasonography, are not routinely incorporated into the clinical framework for pediatric brain death evaluation. In this report, we describe the case of a Japanese child with probable brain death, in whom both TCD and MRI revealed an absence of cerebral perfusion, providing valuable comparative data.

A four-year-old girl with influenza A who developed recurrent seizures was diagnosed with influenza encephalopathy. Despite intensive treatment, no neurological improvement was observed, and she finally met the Japanese criteria for probable brain death.

TCD, performed via bilateral temporal windows using a Phillips EPIQ Elite ultrasound system (Philips Healthcare, Sydney, New South Wales, Australia) equipped with an S5-1 probe, revealed no intracranial blood flow, even at a minimum color Doppler velocity of 1.4 cm/s. Consistently, preliminary MRI, including magnetic resonance angiography (MRA) and arterial spin labeling (ASL), had revealed an absence of cerebral perfusion.

This is the first Japanese pediatric report directly comparing TCD and MRI with ASL in a case of probable brain death. The concordant absence of cerebral perfusion provides evidence for the diagnostic reliability of TCD as a potential adjunctive tool in Japan, in which, although not legally required, ancillary testing may aid clinical assessment and family discussions.

## Introduction

The definition of brain death differs internationally. However, despite efforts toward global harmonization, no standardized tests or ancillary studies currently exist. Although large international initiatives, such as the World Brain Death Project, have proposed minimum clinical standards to reduce this global variability [1], surveys continue to reveal substantial differences among different countries regarding the diagnostic criteria [2]. In Japan, brain death is legally diagnosed only when organ transplantation is considered, whereas otherwise, the condition is termed “probable brain death” rather than assigning a formal diagnosis. This distinction reflects Japan's unique legal and ethical considerations regarding end-of-life care.

Ancillary tests are used to support the diagnosis of brain death by demonstrating the absence of cerebral perfusion when clinical assessment alone is insufficient or inconclusive. Transcranial Doppler (TCD) ultrasonography, which uses a low-frequency probe to evaluate intracranial blood flow, serves as an adjunctive tool to identify cerebral circulatory arrest and has the advantage of being readily performed at the bedside. Arterial spin labeling (ASL) provides a non-invasive magnetic resonance-based assessment of cerebral perfusion that does not require contrast agents or technically demanding procedures such as cerebral angiography, thereby offering additional support for confirming absent cerebral blood flow.

Although international reports have described the use of TCD in pediatric patients with brain death [1,3], its application in Japan remains limited, and domestic data are scarce [4]. In this case study, we present the results of a TCD examination performed on a Japanese child diagnosed with probable brain death, including a clarification of the acoustic window used, and discuss its potential application in Japan, in which ancillary tests are sometimes conducted to complement clinical criteria. Notably, we were also able to undertake a direct comparison of the TCD findings with those of MRI perfusion imaging.

## Case presentation

**Figure 1:**
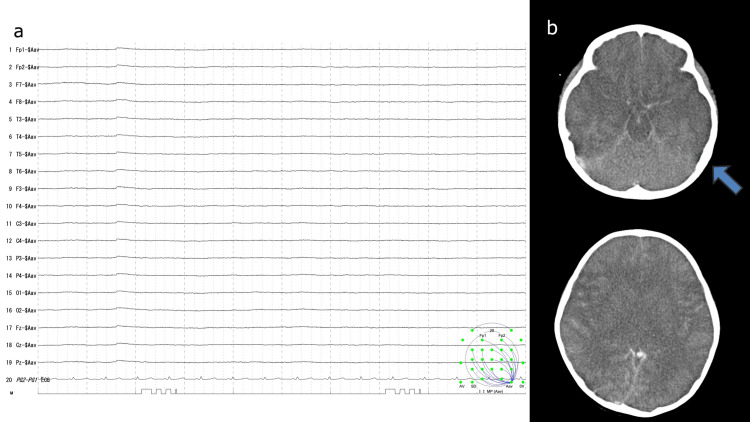
a. The electroencephalogram (EEG) was flat. b. The head CT scan revealed severe cerebral edema.

A four-year-old girl with no previously reported health issues, who presented to a local clinic with a fever, was diagnosed with influenza A. The patient subsequently developed recurrent seizures and was transferred to the emergency department of our hospital. Given her state of altered consciousness (Glasgow Coma Scale E1V1M2), she was admitted to the pediatric intensive care unit, wherein she was diagnosed with influenza encephalopathy. The treatment regimens included steroid pulse therapy, mitochondrial cocktail, peramivir, fosphenytoin, mechanical ventilation, vasopressors, plasma exchange, and normothermia. However, despite these interventions, her condition failed to improve. On the second day of hospitalization, she developed a flat electroencephalogram (EEG), and a head CT revealed significant cerebral edema, characterized by loss of grey-white matter differentiation, effacement of the cerebral sulci, and compression of the ventricular system (Figure 1). On the seventh day, vasopressor medication was discontinued, and no evidence of neurological recovery was observed thereafter. By the 10^th^ day, her condition met the Japanese criteria for probable brain death, characterized by a deep coma, fixed dilated pupils, an absence of brainstem reflexes, and a flat EEG.

**Figure 2:**
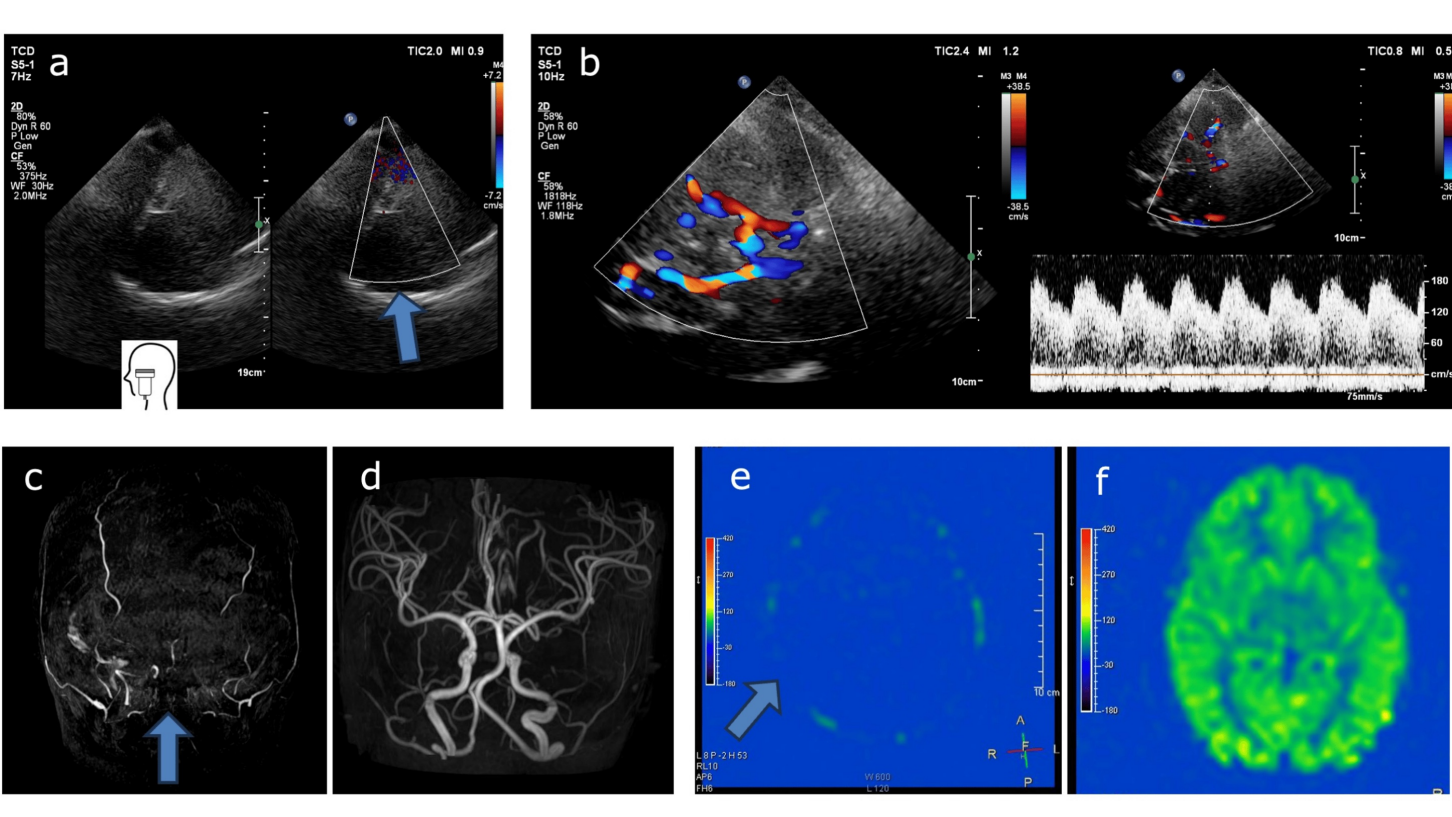
Comparison with normal findings in Transcranial Doppler (TCD) examination and MRI a. Even on reducing the color Doppler velocity to the minimum value, no blood flow was detected bilaterally. b. Representative normal TCD in another case. c. No blood flow was observed on magnetic resonance angiography (MRA). d. Representative normal MRA in another case. e. No blood flow was detected on arterial spin labeling (ASL). f. Representative normal ASL in another case.

On the 37^th^ day, TCD was conducted via the bilateral temporal acoustic windows, using a Philips EPIQ Elite series ultrasound device (Philips Healthcare, Sydney, New South Wales, Australia) equipped with an S5-1 probe (4 Hz) (Figure 2a). Despite reducing the color Doppler velocity to a minimum value of 1.4 cm/s, no blood flow was detected bilaterally in the middle or posterior cerebral arteries. Consistently, an absence of perfusion had been observed on magnetic resonance angiography (MRA), and ASL performed on the 14^th^ day (Figures 2c, 2e). MRI was performed using a 3.0-T scanner. The acquisition parameters for three-dimensional ASL were as follows: repetition time/echo time (TR/TE), 8000/11.0 ms; post-labeling delay, 1500 ms; field of view, 240 × 240 mm; slice thickness, 6 mm; number of excitations, 4; number of slices, 14; and total acquisition time, 5 minutes 4 seconds. These findings, obtained at separate time points using different modalities during a continuous loss of brain function, revealed a consistent absence of intracranial circulation. Since these results are not typical for children, the normal results obtained using the same equipment with other healthy children are shown (Figures 2b, 2d, 2f).

## Discussion

We present the case of a Japanese child with probable brain death, in whom an absence of cerebral blood flow was detected using both TCD and MRI.

The use of TCD in brain death evaluation differs among different countries. In Japan, the 2011 criteria for brain death did not include the use of TCD, and even in the 2024 revision of the guidelines, TCD was not advocated as an ancillary assessment [5]. Conversely, although the 2023 U.S. guidelines permit the use of TCD as an adjunctive test in adults, owing to insufficient validation in the pediatric population, this use does not extend to children [1]. However, although the American College of Radiology recommends against the routine use of TCD, it does note that it may still serve as an adjunctive option in certain circumstances [3]. Nevertheless, the findings of a meta-analysis of adults indicated that repeated TCD may assist in determining the timing of angiography, which remains the gold standard for confirming the arrest of cerebral circulation [6]. Although certain TCD flow patterns have been described as suggestive of cerebral circulatory arrest, in approximately 10% of cases, detectable signals are absent. Moreover, the results may be influenced by operator skills, cardiac output, hematocrit, and PaCO₂, and, consequently, at present, TCD cannot be considered a gold standard [7]. However, the ability to perform bedside examinations without transferring the patient to a dedicated imaging suite is a major advantage compared with other ancillary procedures. Reports from Japan have also described the potential utility of TCD in this context, emphasizing the value of repeated examinations [4].

Specific challenges in pediatric TCD include the potential for misinterpretation due to fontanel compression in infants, which can reduce systolic flow [8]. In addition, a reversal of diastolic flow in children with congenital heart disease and extracardiac shunts may lead to misinterpretation [9].

In the case reported herein, the confirmation of an absence of cerebral perfusion using both TCD and MRI with ASL is particularly noteworthy, as MRA alone is not considered sufficiently reliable in the 2023 U.S. guidelines. The capacity to compare these modalities within the same clinical course contributes to validating the reliability of the findings and adds reporting value, particularly in a Japanese context. However, whereas MRI has been reported to lack sensitivity in detecting early or minimal cerebral blood flow, combining this imaging with ASL can contribute to enhancing diagnostic confidence in identifying cerebral circulatory arrest [5, 7]. To the best of our knowledge, this is the first Japanese pediatric report that directly compares the findings of TCD with those of MRI used in conjunction with ASL in a case of probable brain death. Such comparative data are important in Japan, where, although ancillary testing is not legally mandated, it is often used to support clinical assessment and facilitate family discussions. As this is a single case report, our findings should be interpreted with caution. Further studies involving larger cohorts are necessary to evaluate the reliability and generalizability of this approach.

## Conclusions

In the pediatric patient described herein, we confirmed the absence of cerebral perfusion not only by performing TCD but also using MRI, which accordingly makes it noteworthy. The direct comparison between TCD and MRI perfusion imaging enhances the significance of this case. TCD is a simple bedside test frequently used internationally and may have potential application as an adjunct test for determining brain death in Japanese children. Further case studies in Japan are required to enable a more comprehensive assessment of its utility.
